# Comparison of Thiamin Diphosphate High-Performance Liquid Chromatography and Erythrocyte Transketolase Assays for Evaluating Thiamin Status in Malaria Patients without Beriberi

**DOI:** 10.4269/ajtmh.20-0479

**Published:** 2020-09-28

**Authors:** Andrew J. Taylor, Dinesh Talwar, Sue J. Lee, Lorna Cox, Mayfong Mayxay, Paul N. Newton

**Affiliations:** 1 Lao Oxford-Mahosot Hospital-Wellcome Trust Research Unit (LOMWRU), Microbiology Laboratory, Mahosot Hospital, Vientiane, Lao PDR;; 2 Centre for Tropical Medicine and Global Health, Churchill Hospital, University of Oxford, Oxford, United Kingdom;; 3 Scottish Trace Element and Micronutrient Diagnostic and Research Laboratory, Glasgow Royal Infirmary, Glasgow, United Kingdom;; 4 Mahidol-Oxford Tropical Medicine Research Unit, Faculty of Tropical Medicine, Mahidol University, Bangkok, Thailand;; 5 MRC Elsie Widdowson Laboratory, Cambridge, United Kingdom;; 6 Institute of Research and Education Development, University of Health Sciences, Ministry of Health, Vientiane, Lao PDR

## Abstract

Thiamin deficiency, or beriberi, is an increasingly re-recognized cause of morbidity and mortality in the developing world. Thiamin status has traditionally been measured through the erythrocyte activation assay (ETKA) or basal transketolase activity (ETK), which indirectly measure thiamin diphosphate (TDP). Thiamin diphosphate can also be measured directly by high-performance liquid chromatography (HPLC), which may allow a more precise estimation of thiamin status. We compared the direct measurement of TDP by HPLC with basal ETK activity and ETKA in 230 patients with *Plasmodium falciparum* malaria in rural southern Laos without overt clinical beriberi, as part of a trial of thiamin supplementation. Admission thiamin status measured by basal ETK activity and ETKA (α) were compared with thiamin status assessed by the measurement of TDP by HPLC. 55% of 230 included patients were male, and the median age was 10 (range 0.5–73) years. Using α ≥ 25% as the gold standard of thiamin deficiency, the sensitivity of TDP < 275 ng/gHb as a measure of thiamin deficiency was 68.5% (95% CI: 54.4–80.5%), with specificity of 60.8 (95% CI: 53.2–68.1%). There was a significant inverse correlation between the results of the two tests (Kendall’s tau = −0.212, *P* < 0.001). Basal ETK activity was also significantly positively correlated with TDP levels (Kendall’s tau = 0.576, *P* < 0.001). Thiamin diphosphate measurement may have a role in measuring thiamin levels in clinical settings. Further studies evaluating TDP concentration in erythrocytes with basal ETK activity and ETKA (α) in beriberi patients would help establish comparative values of these assays.

## INTRODUCTION

Until the 1950s, thiamin (vitamin B1, thiamine) deficiency was recognized as an important healthcare problem in the developing world with much public health research and debate.^1^ Incidence has since declined in wealthier areas, but there are increasing reports of infantile and adult thiamin deficiency in less wealthy areas of the world, especially in Asia.^2–6^ The symptoms and signs of thiamin deficiency, or beriberi, include myocardial dysfunction, encephalopathy, and peripheral neuropathy, and may be fatal. However, beriberi is readily and inexpensively treatable with parenteral thiamin. Diagnosis of thiamin deficiency is almost always clinical, which is possible in patients with clear deficiency, but is more challenging in those with less marked symptoms or with concurrent infections. Clinically unapparent thiamin deficiency has been shown to be common among sick infants admitted to hospital in Vientiane^2^ and in Cambodian infants.^4^

Thiamin status has traditionally been assessed by indirect, functional measurement of thiamin diphosphate (TDP) in erythrocytes using the transketolase activation assay.^7^ This calculates the activation coefficient (α) by measuring the erythrocyte transketolase activity (ETK) before (basal) and after (activated) the addition of TDP in vitro. The transketolase activation assay, however, is limited by the expense and rare availability of the test, reference ranges which have not been standardized in children or by geographical regions,^7–9^ poor inter-assay replicability, sample storage instability due to rapid inactivation of the transketolase enzyme, and no external quality assurance systems.^10^ Furthermore, it has been demonstrated that the apoenzyme may not reactivate following chronic deficiency in vivo,^10,11^ binding of the apoenzyme and coenzyme may be altered by the presence of isoenzymes or other cofactors (e.g., magnesium),^12^ and synthesis may be reduced in those with diabetes and liver disease.^13,14^ Recently, it has been suggested that basal erythrocyte ETK activity is a more accurate biomarker of infantile beriberi than the activation coefficient,^3^ although there are no recognized reference ranges established for basal ETK activity.

An alternative method for the evaluation of thiamin status is the direct measurement of erythrocyte TDP by high-performance liquid chromatography (HPLC).^15^ Thiamin diphosphate concentrations in erythrocytes are good indicators of body stores,^16^ and the method has been shown to be accurate and easier to be standardized than ETKA.^15,17^ However, the TDP method shares the disadvantages of ETKA (α) in terms of having reference ranges that are not standardized in children or by geographical region. Current reference ranges for TDP are descriptive of a “normal population” and not based on physiology. Furthermore, the relationship between ETKA and TDP (α) is not clear.

Data for this study come from a trial of malaria supplementation in southern Laos.^18^ Malaria is a common cause of morbidity in southern Laos, and one third of patients present with biochemical thiamin deficiency.^18^ It was postulated thiamin supplementation may improve neurological side effects and adverse outcomes.^5,18^ The trial demonstrated that thiamin levels increased following supplementation, but there was no reduction in adverse events. Here, we compare the assessment of thiamin status by direct measurement of TDP using HPLC against erythrocyte transketolase assays in these malaria patients.^18^

## METHODS

### Patient cohort.

We compared admission TDP concentration with ETKA (α) and basal ETK activity in erythrocytes in 230 patients infected with *Plasmodium falciparum*, as demonstrated by positive admission Giemsa-stained peripheral blood slides, in southern Laos; see ref. 18 for details. Samples were collected during the rainy season, when malaria transmission is high, from June to November 2008 to 2010. None of the patients had documented, clinically apparent, thiamin deficiency and none were known to have received thiamin supplementation. No patients were fasted. Written informed consent was obtained from all participants. Ethical clearance for the study was granted by the Lao National Ethics Committee for Health Research and the Oxford University Tropical Medicine Research Ethics Committee.

### Blood sample preparation.

Immediately after venous blood collection, 1 mL lithium heparin anticoagulated blood was centrifuged and washed in phosphate-buffered saline three times, with removal of the buffy coat initially and again after each wash. Washed erythrocyte samples were stored at −30°C for maximum of 3 months and then at −80°C until shipment to the United Kingdom on dry ice.

### Erythrocyte transketolase activity assays.

Erythrocyte transketolase activity assays were performed at the Medical Research Council Elsie Widdowson Laboratory in Cambridge, United Kingdom, using an adaptation of the method of Vuilleumier et al.^19^ as described in Khounnorath et al.^2^ and Soukaloun et al.^3^ These are functional assays for the thiamin-dependent erythrocyte transketolase enzyme in washed red cells. The assays were performed in microplate format in a temperature-controlled instrument which provided a consistent temperature over the whole plate (Multiskan, Thermo Fisher Scientific, Waltham, MA); 20 samples and three single-donor washed erythrocyte quality controls (QC), prepared in-house, were assayed on each plate. All QC materials were obtained from persons with adequate thiamin status; no “deficient” samples were available. Hemoglobin (Hb) was measured in the same lysate using Zap-o-globin (Beckman-Coulter, Brea, CA).

The activation coefficient in vitro may be expressed either as the ratio of ETK activity after activation with excess TDP to basal ETK activity or as α. α is the ratio of in vitro ETK activity after TDP has been added minus the basal ETK activity before TDP has been added, to the basal ETK activity, expressed as a percentage. Expressing ETKA as α greatly magnifies the discrimination. Higher ETKA ratio or α represents a greater degree of thiamin deficiency. The threshold for biochemical thiamin insufficiency is regarded as a ratio > 1.25 or its equivalent, α = 25. In this study, thiamin deficiency was defined as α ≥ 25.^18^ Basal ETK activity of 0.59 per g/Hb was used as a cutoff value to signify biochemical deficiency.^3^ The between-batch imprecision of the assay was 14–20% for ETKA expressed as α (3–5% when expressed as ratio), and 7–10% for ETK/gHb.

### Thiamin diphosphate measurement.

Thiamin diphosphate measurement was performed at the Scottish Trace Element and Micronutrient Diagnostic and Research Laboratory. Erythrocytes were prepared as described for the transketolase assay, and TDP was measured by HPLC using post-column derivation with alkaline potassium ferricyanide as described in the study by Talwar et al.^15^ The same blood was drawn, but different aliquots were used for the two assays. Internal QC samples were used; QC1 was hemolysate, and QC2 was prepared by addition of TDP solution to the hemolysate. Six QCs were run with each batch of 30 samples: two at the start, two in the middle, and two at the end of the run. A lower limit of the adult reference range of 275 ng/gHb TDP was used based on the lower 2.5 percentile of concentrations found in Talwar’s “normal” population.^15^ This value was not based on physiological considerations or requirements for thiamin. The between-batch CV of the assay was < 7%.

### Statistical analysis.

Graphs were plotted comparing erythrocyte TDP concentrations with ETKA (α) and basal ETK activity. Kendall’s rank correlation coefficient (τ) was calculated to assess the association between the continuous values of TDP, ETKA (α), and basal ETK activity. Sensitivity and specificity of TDP ≥ 275 ng/gHb were calculated using α ≥ 25% as the gold standard, as this has been the established method for measuring thiamin status. “Ideal” TDP cutoffs were estimated using α ≥ 25% and basal ETK activity < 0.59 as the reference variables to indicate those with “true” thiamin deficiency. Receiver operating characteristic (ROC) curves were plotted, and the “ideal” cutoff was defined as the point closest to the top left corner of the graphs.

## RESULTS

Samples from 230 patients were included, of whom 55% were male. The median age was 10 (range 0.5–73) years, with 78 patients aged > 16 and 152 patients aged < 16 years.

Of 230 patients, 106 had erythrocyte TDP levels < 275 ng/gHb. Of these, 34.9% (37/106) also had α levels ≥ 25%, whereas 65.1% (69/106) had α levels < 25%. One hundred twenty-four patients had erythrocyte TDP levels ≥ 275 ng/gHb, of which 86.3% (107/124) also had α levels < 25% and 13.7% (17/124) had α levels ≥ 25%. Fifty-four patients had α levels ≥ 25%, of which 68.5% (37/54) also had erythrocyte TDP levels < 275 ng/gHb (Table 1).

**Table 1:** Frequency of thiamin deficiency using admission α ≥ 25% and erythrocyte TDP < 275 ng/gHb in 230 malaria patients

	TDP < 275	TDP ≥ 275	Total
α ≥ 25%	37	17	54
α < 25%	69	107	176
Total	106	124	230

TDP = thiamin diphosphate.

Using α ≥ 25% as the gold standard of biochemical thiamin deficiency, the sensitivity of erythrocyte TDP < 275 ng/gHb as a measure of thiamin deficiency was 68.5% (95% CI: 54.4–80.5%) with specificity of 60.8 (95% CI: 53.2–68.1%). There was a significant inverse correlation between the tests (Kendall’s tau = −0.212, *P* < 0.001). Inverse correlation was also observed between the two assays when adults (Supplemental Figure 2) (78 patients, Kendall’s tau = −0.227, *P* = 0.003) and children (Supplemental Figure 1) (152 patients, Kendall’s tau = −0.176, *P* = 0.001) were analyzed separately.

Basal ETK activity was significantly positively correlated with erythrocyte TDP levels (Kendall’s tau = 0.576, *P* < 0.001, Figure 2, Table 2, Supplemental Figure 3). Of 106 patients with TDP levels < 275 ng/gHb, 98.1% (104/106) had basal ETK activity < 0.59 μmoles/minute/gHb. Of 179 patients with a basal ETK activity < 0.59 μmoles/minute/gHb, 58.1% (104/179) had TDP levels < 275 ng/gHb. A correlation was also demonstrated when adults (78 patients, Kendall’s tau = 0.597, *P* < 0.001) and children (152 patients, Kendall’s tau = 0.546, *P* < 0.001) were analyzed separately. Basal ETK was significantly lower in adults than in children aged < 16 years (0.44 versus 0.52, *P* = 0.003), as was TDP (265 versus 314, *P* = 0.003).

**Figure 1.**
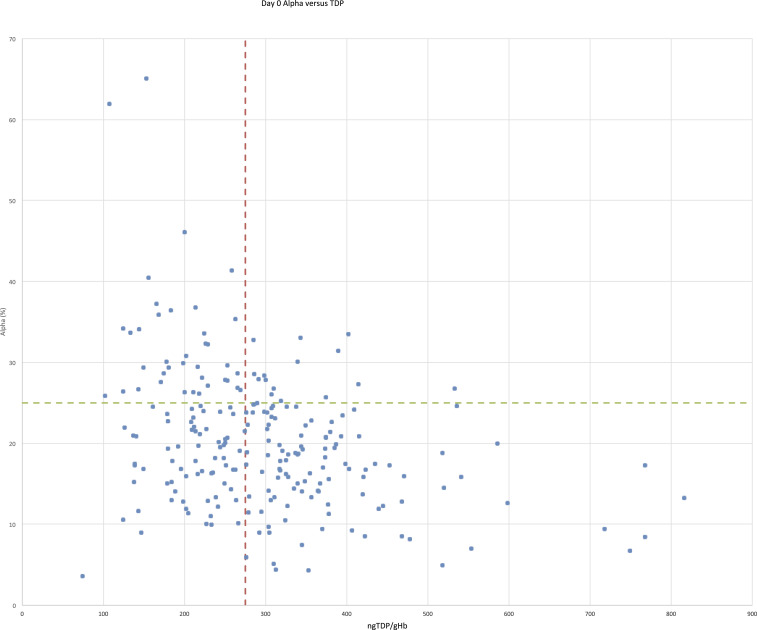
Relationship between admission alpha and thiamin diphosphate for 230 malaria patients. This figure appears in color at www.ajtmh.org.

**Figure 2.**
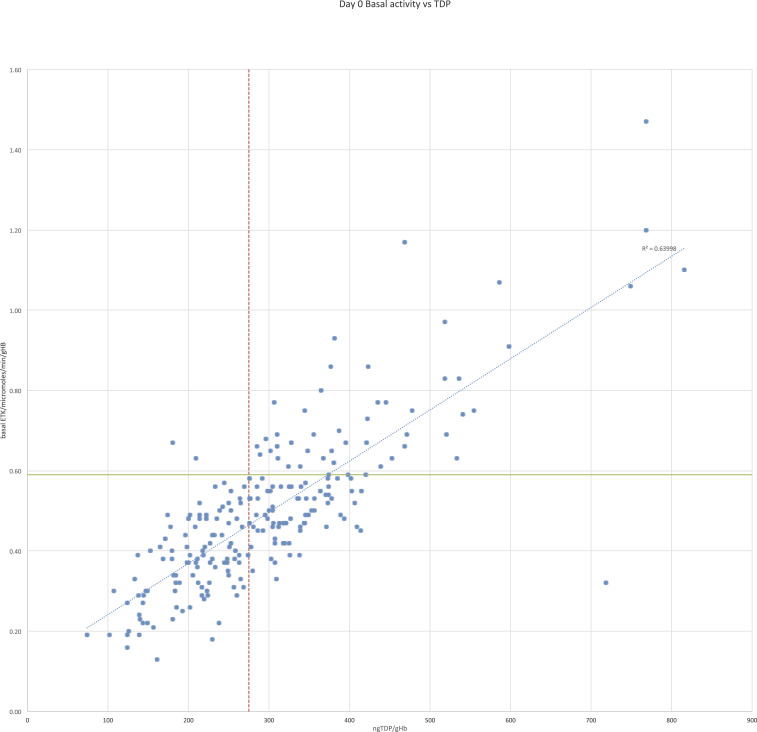
Relationship between admission basal ETK and thiamin diphosphate for 230 malaria patients. This figure appears in color at www.ajtmh.org.

**Table 2:** Frequency of admission basal erythrocyte ETK activity < 0.59 μmoles/minute/gHb and TDP concentration < 275 ng/gHb for 230 malaria patients

	TDP < 275	TDP ≥ 275	
Basal ETK activity < 0.59	104	75	179
Basal ETK activity ≥ 0.59	2	49	51
	106	124	230

TDP = thiamin diphosphate.

Nonparametric ROC curves estimated that the optimum erythrocyte TDP value for α ≥ 25% prediction was < 269 ng/gHb, which gave a sensitivity and specificity of 68.5% and 61.4%, respectively; this resulted in 63.0% of patients being correctly classified (area under the ROC [AUROC] curve = 0.6820). For basal ETK activity < 0.59 μmoles/minute/gHb, a TDP cutoff of < 321 ng/gHb gave a sensitivity of and specificity of 78.8% and 80.4%, respectively, and resulted in 79.1% of patients being correctly classified (AUROC curve = 0.8881).

## DISCUSSION

This study compared erythrocyte TDP concentration with ETKA (α) to evaluate biochemical thiamin status in malaria patients without clinical beriberi. Results showed that TDP concentration in erythrocytes was inversely correlated with ETKA (α) in both adults and children. When comparing the tests’ ability to define biochemical thiamin deficiency (comparing erythrocyte TDP < 275 ng/gHb with α levels > 25%), TDP had a sensitivity of 68.5% (95% CI: 54.4–80.5%) and a specificity of 60.8% (95% CI: 53.2–68.1%). The correlation between basal ETK activity and erythrocyte TDP concentration was stronger than the correlation between α and erythrocyte TDP (0.576 versus −0.212, respectively, *P* < 0.001).

Both ETKA and TDP assays have limitations. Thiamin diphosphate is not a metabolic functional endpoint and does not determine whether the thiamin concentration present is sufficient to saturate the enzyme. Furthermore, the lower reference range of erythrocyte TDP (275 ng/gHb) used in this study was not based on physiological considerations or human requirements for thiamin. ETKA may therefore have a theoretical advantage in measuring thiamin status, as it is a functional measure.

Limitations of this study include the fact that no patients in the cohort manifested clinical signs of thiamin deficiency, so it is not possible to correlate results with clinical thiamin deficiency. It is possible that some patients had subclinical deficiency. Determining a clinical endpoint for thiamin deficiency is challenging, as beriberi seems to occur not only when thiamin deficiency exists but also when additional exacerbating factors such as infections are present. The distinction between those who do and do not show clinical symptoms of beriberi at different biochemical assay levels may also be confounded by other factors. Importantly, these data cannot be used to inform biochemical diagnosis of infantile beriberi as children aged < 6 months were not included.

Further work is required to understand the relationship between erythrocyte TDP levels and ETKA with clinical features of beriberi and patient outcomes. Measurement of TDP in erythrocytes showed a clear linear correlation with basal ETK activity (Figure 2), suggesting it may have a role in measuring thiamin status. Further studies comparing TDP concentration, basal ETK activity, and ETKA (α) in patients with and without beriberi, before and after thiamin supplementation, would help define reference levels for biochemical thiamin deficiency for different patient ages and establish the comparative values of these assays.

## Supplemental material

Supplemental materials
