# Supplementary figures and images for: Comparison of Thiamin Diphosphate High-Performance Liquid Chromatography and Erythrocyte Transketolase Assays for Evaluating Thiamin Status in Malaria Patients without Beriberi
Source: Am J Trop Med Hyg. 2020 Sep 28;103(6):2600–4. doi: 10.4269/ajtmh.20-0479 (PMC7695103; doi:10.4269/ajtmh.20-0479)

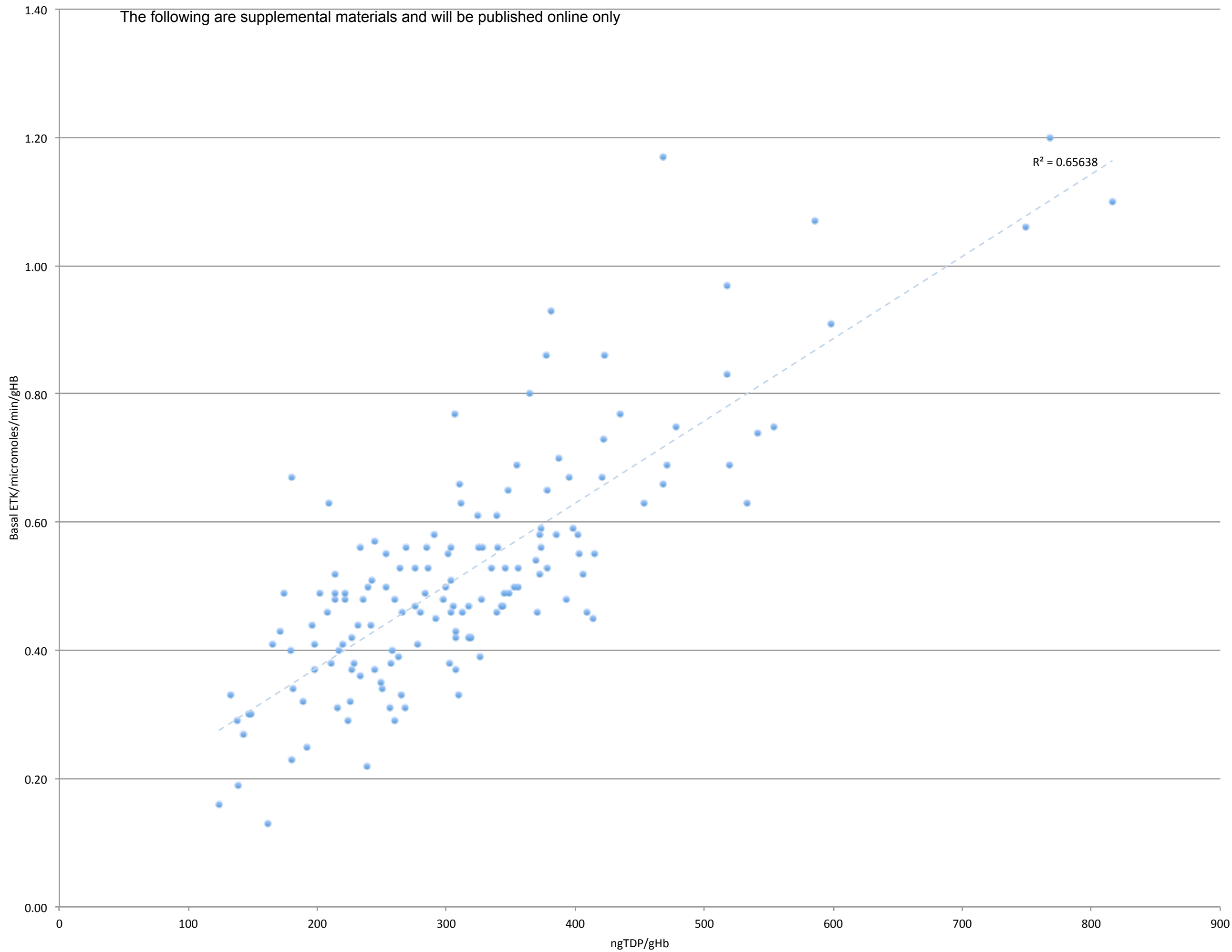

Supplement: Supplementary file 1 [file tpmd200479.SD1.pdf]

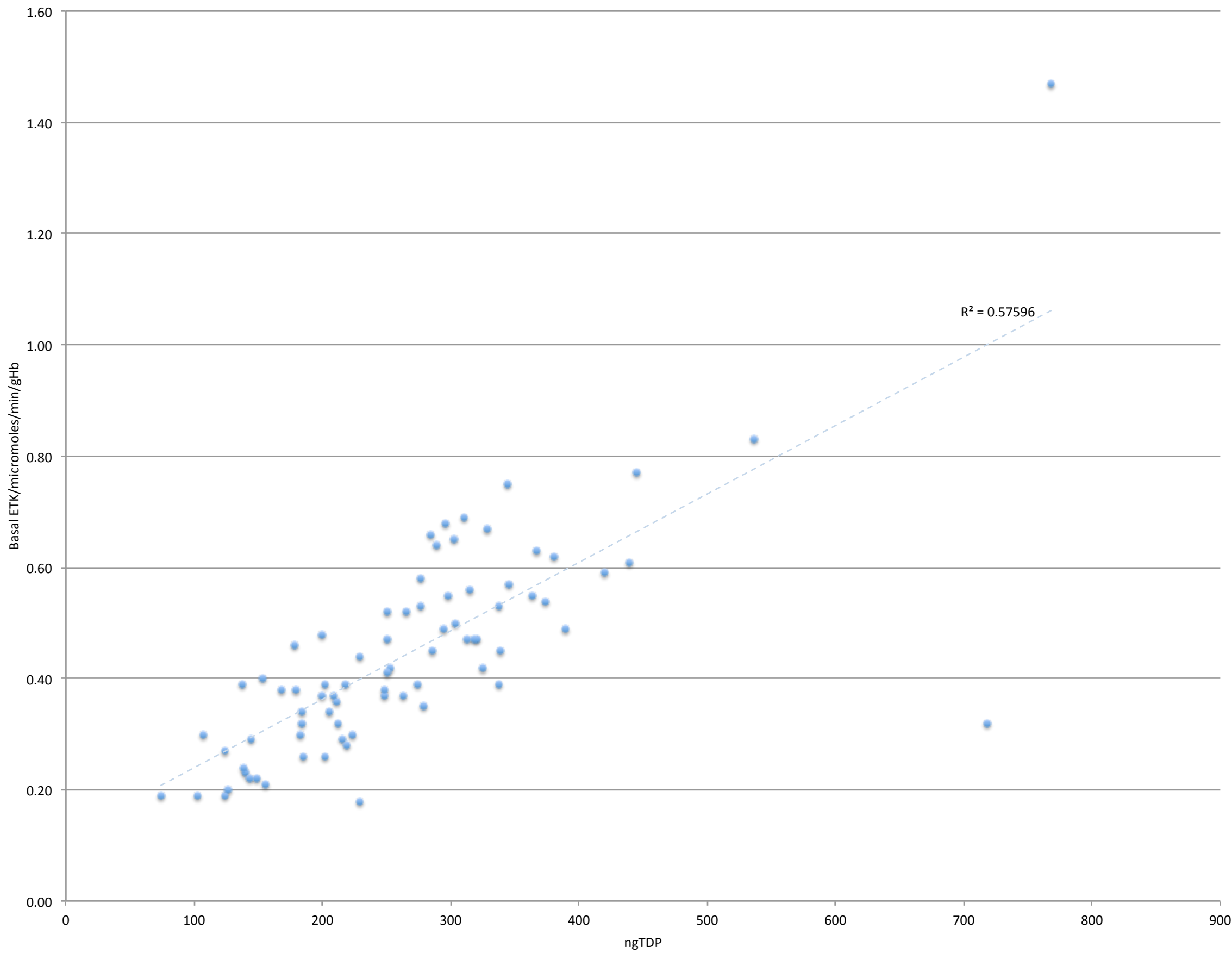

Supplement: Supplementary file 2 [file tpmd200479.SD2.pdf]

# ngTDP/gHb vs Day 0 basal activity in children and adults

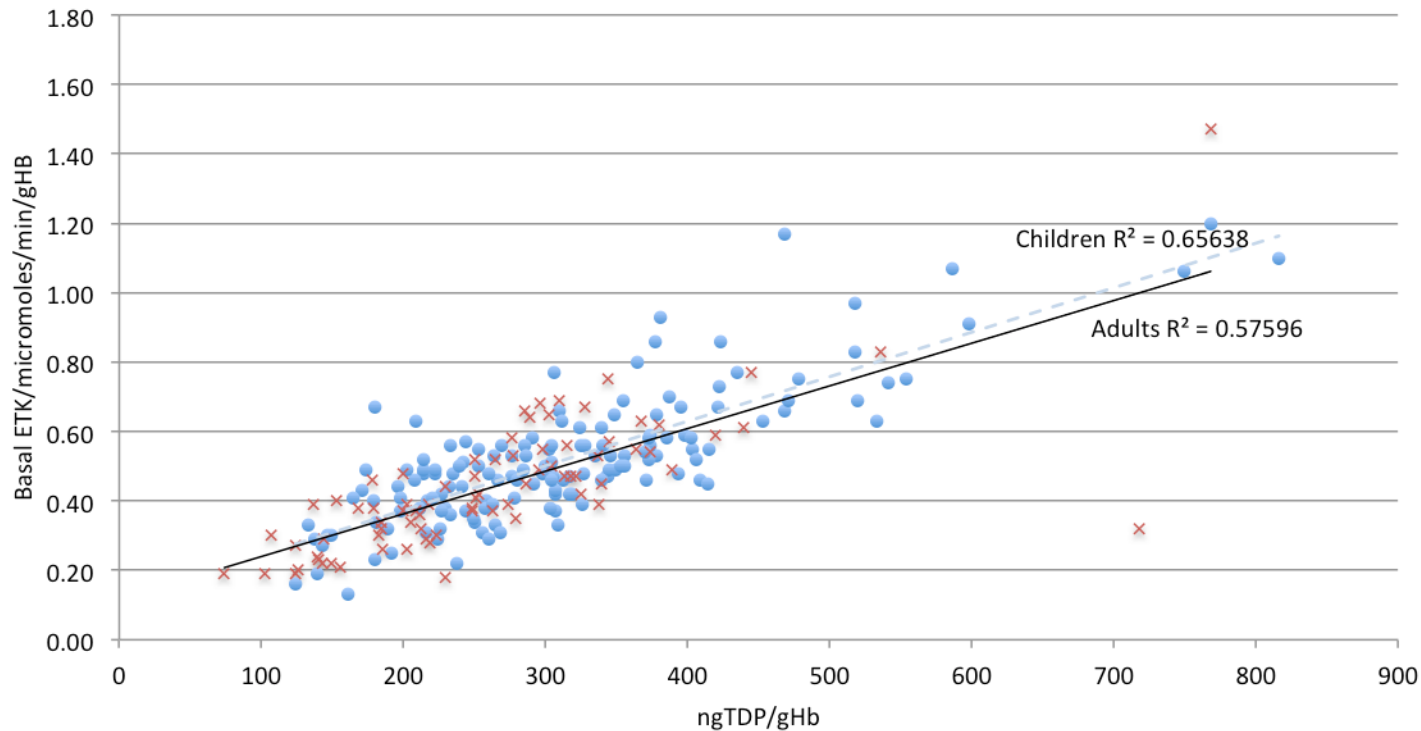

Supplement: Supplementary file 3 [file tpmd200479.SD3.pdf]

Day 0 Activity co-efficient (activated/basal activity) vs tdp

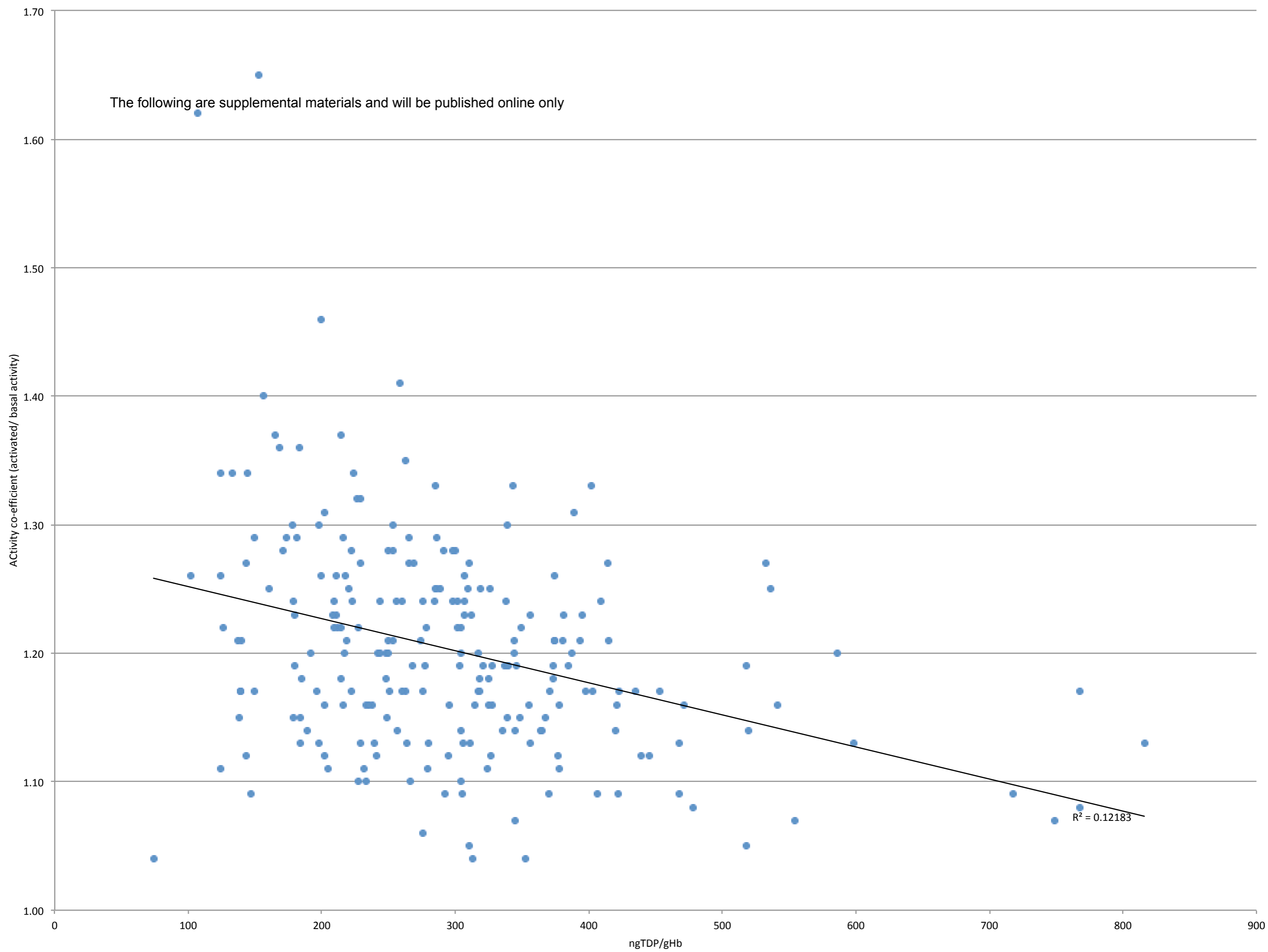

Supplement: Supplementary file 4 [file tpmd200479.SD4.pdf]
