# Supplementary material for: Comparison of Thiamin Diphosphate High-Performance Liquid Chromatography and Erythrocyte Transketolase Assays for Evaluating Thiamin Status in Malaria Patients without Beriberi
Source: Am J Trop Med Hyg. 2020 Sep 28;103(6):2600–4. doi: 10.4269/ajtmh.20-0479 (PMC7695103; doi:10.4269/ajtmh.20-0479)

The following are supplemental materials and will be published online only

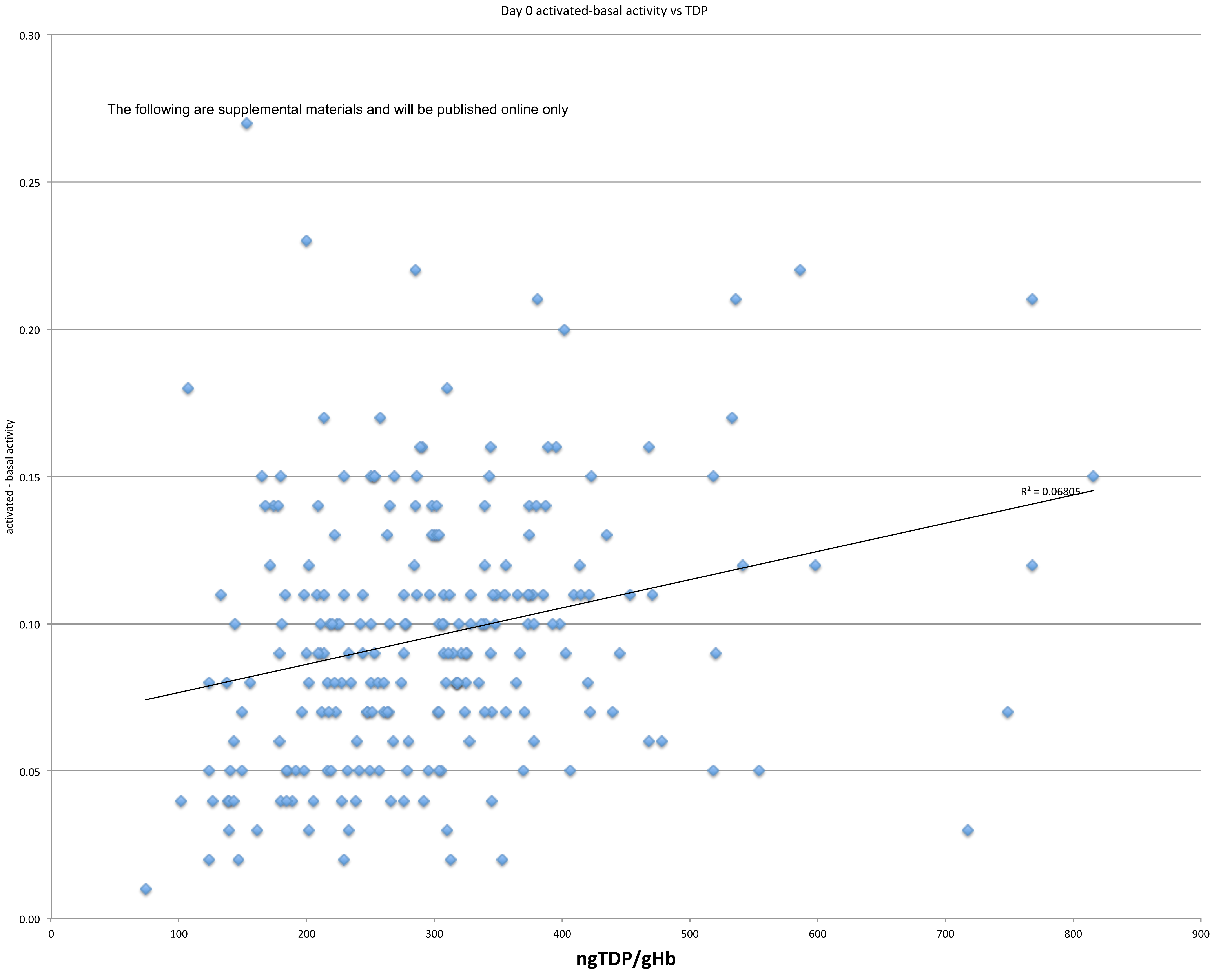

Supplement: Supplementary file 5 [file tpmd200479.SD5.pdf]
