# Supplementary material for: Comparison of Thiamin Diphosphate High-Performance Liquid Chromatography and Erythrocyte Transketolase Assays for Evaluating Thiamin Status in Malaria Patients without Beriberi
Source: Am J Trop Med Hyg. 2020 Sep 28;103(6):2600–4. doi: 10.4269/ajtmh.20-0479 (PMC7695103; doi:10.4269/ajtmh.20-0479)

Supplemental Fig 1: Relationship between Basal ETK and TDP in Children (≤ 16) (n = 152)

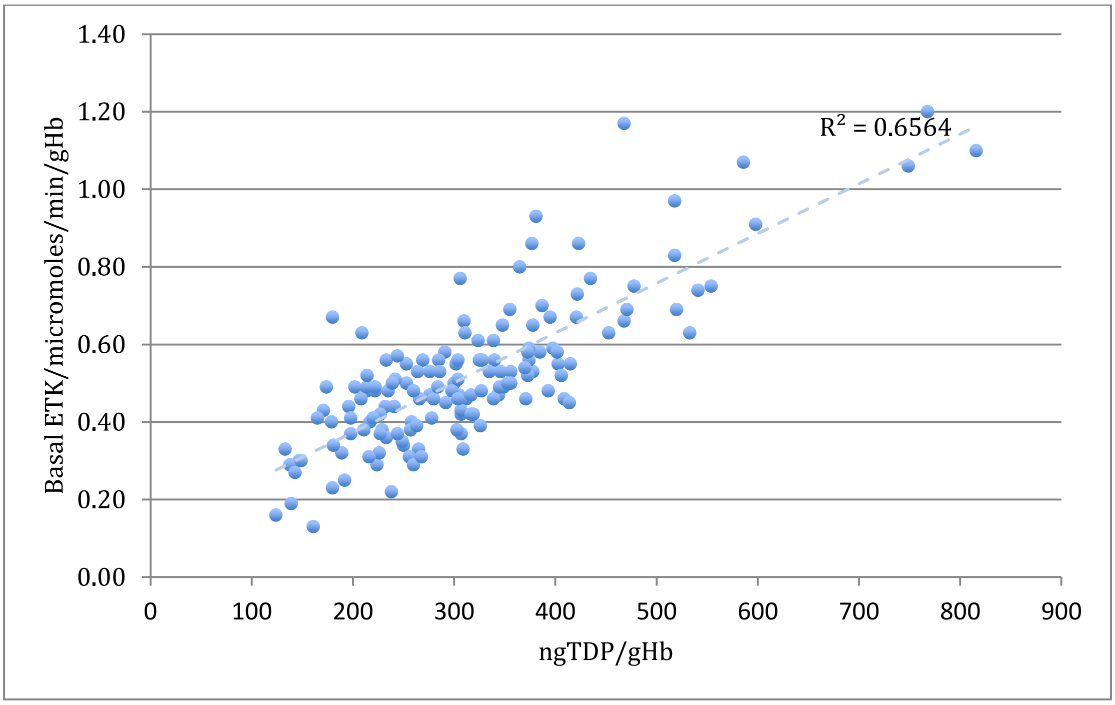

Supplemental Fig 2: Relationship between Basal ETK and TDP in Adults (≤ 16) (n = 78)

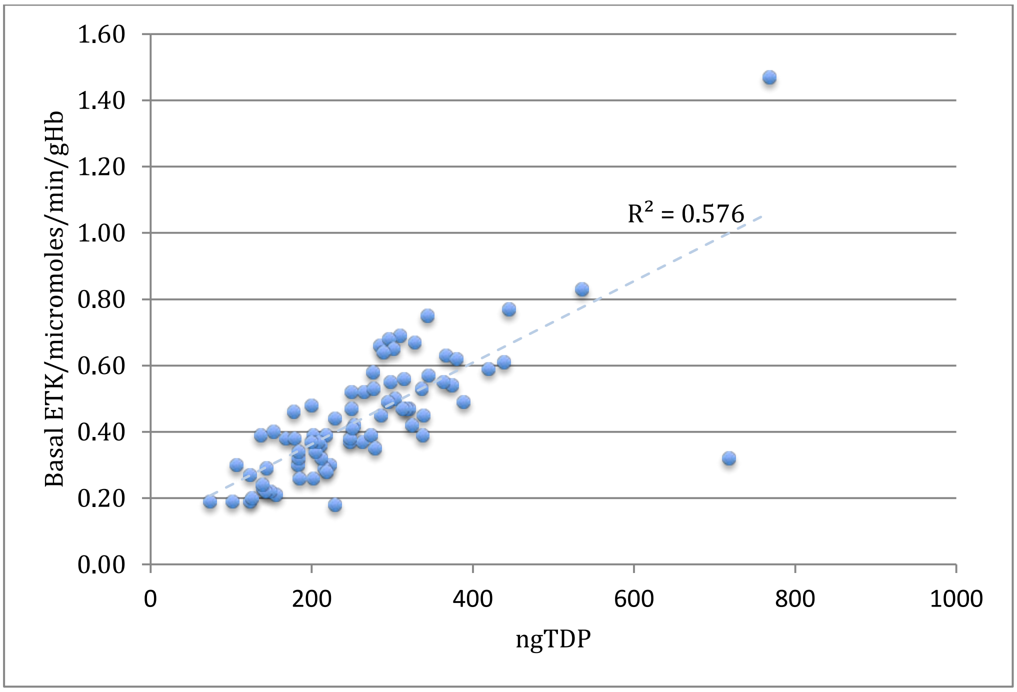

Supplemental Fig 3: Relationship between Basal ETK and TDP in children and adults

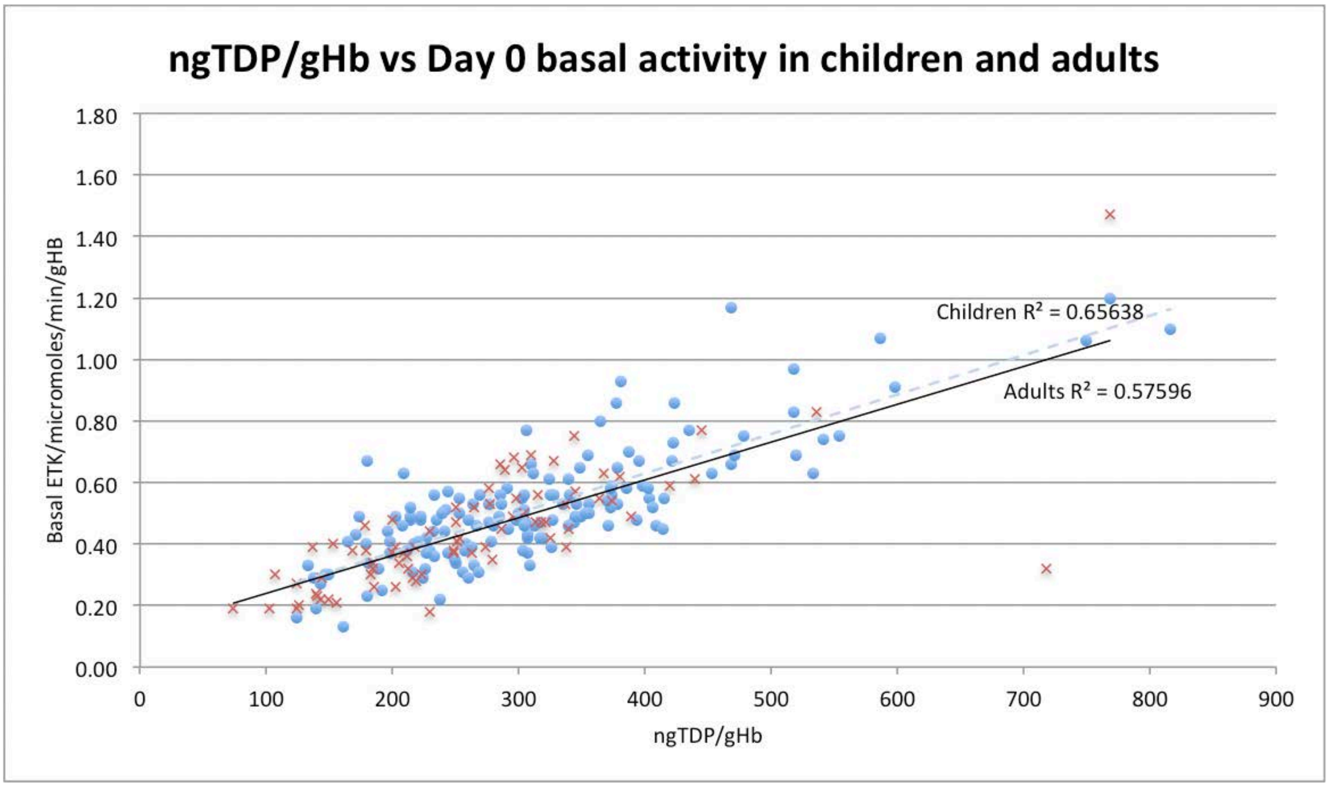

Supplemental Fig 4: Day 0 Activated ETK plotted against TDP.

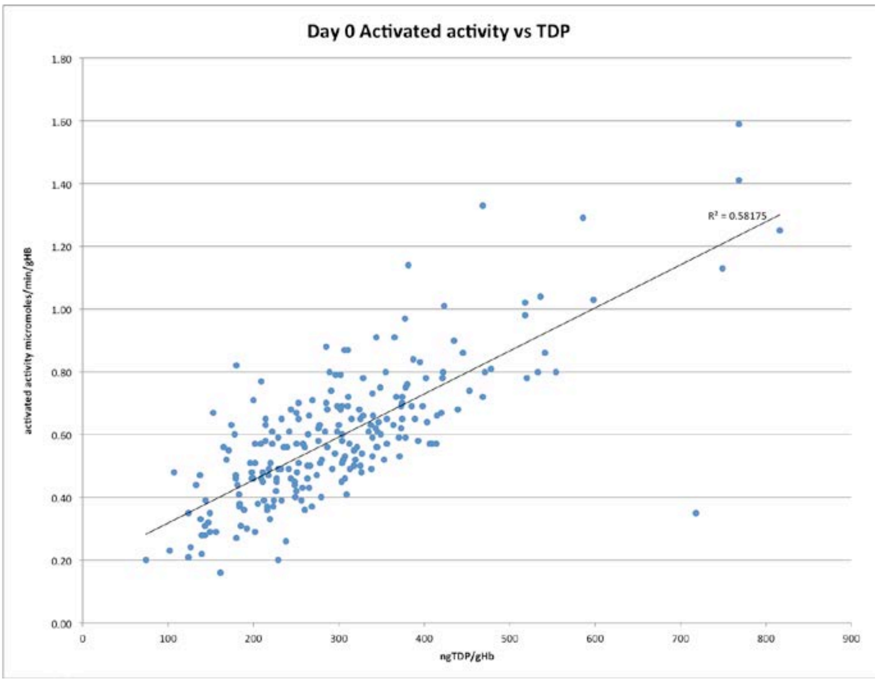

Activated ETK is positively correlated with TDP levels (r = 0.762725) (Supplemental Figure 4). Differences between basal and activated ETK were not shown to be associated with TDP (r = 0.260864) (Supplemental Figure 5).

Supplemental Fig 5: activated-basal ETK activity plotted against TDP

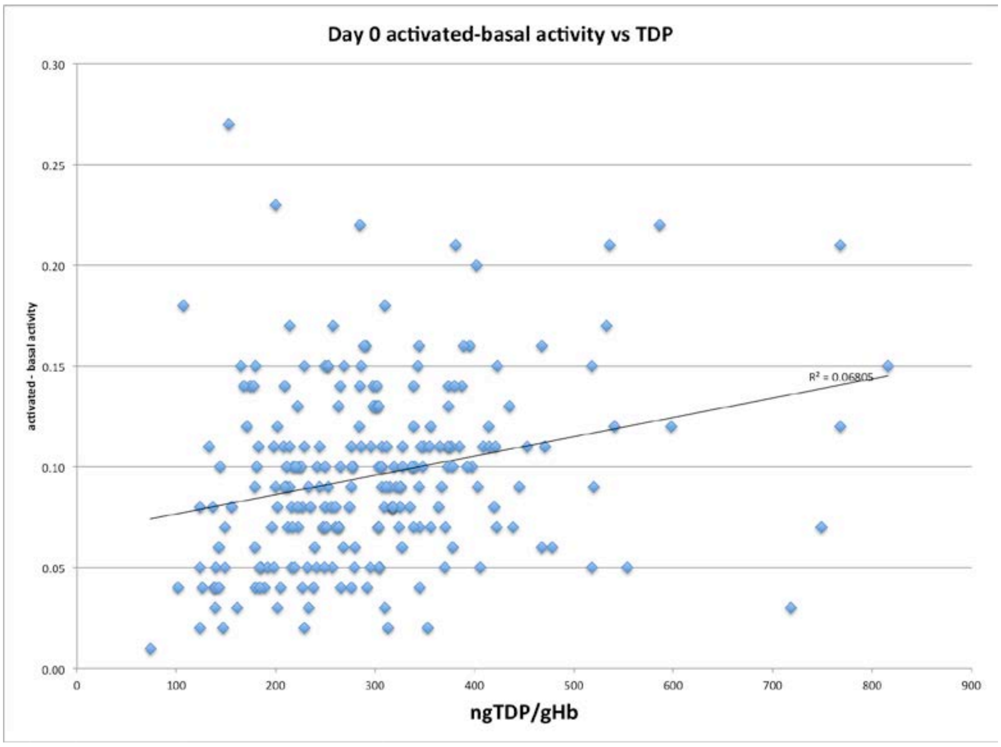

Supplement: Supplementary file 6 [file tpmd200479.SD6.pdf]
